# Extracting time-frequency feature of single-channel vastus medialis EMG signals for knee exercise pattern recognition

**DOI:** 10.1371/journal.pone.0180526

**Published:** 2017-07-10

**Authors:** Yi Zhang, Peiyang Li, Xuyang Zhu, Steven W. Su, Qing Guo, Peng Xu, Dezhong Yao

**Affiliations:** 1 School of Aeronautics and Astronautics, University of Electronic Science and Technology of China, 611731, Chengdu, China; 2 Key Laboratory for NeuroInformation of Ministry of Education, School of Life Science and Technology, University of Electronic Science and Technology of China, 610054, Chengdu, China; 3 Center for Information in BioMedicine, University of Electronic Science and Technology of China, 610054, Chengdu, China; 4 Centre for Health Technologies, Faculty of Engineering and Information Technology, University of Technology, Sydney, NSW 2007, Australia; University of Illinois at Urbana-Champaign, UNITED STATES

## Abstract

The EMG signal indicates the electrophysiological response to daily living of activities, particularly to lower-limb knee exercises. Literature reports have shown numerous benefits of the Wavelet analysis in EMG feature extraction for pattern recognition. However, its application to typical knee exercises when using only a single EMG channel is limited. In this study, three types of knee exercises, i.e., flexion of the leg up (standing), hip extension from a sitting position (sitting) and gait (walking) are investigated from 14 healthy untrained subjects, while EMG signals from the muscle group of vastus medialis and the goniometer on the knee joint of the detected leg are synchronously monitored and recorded. Four types of lower-limb motions including standing, sitting, stance phase of walking, and swing phase of walking, are segmented. The Wavelet Transform (WT) based Singular Value Decomposition (SVD) approach is proposed for the classification of four lower-limb motions using a single-channel EMG signal from the muscle group of vastus medialis. Based on lower-limb motions from all subjects, the combination of five-level wavelet decomposition and SVD is used to comprise the feature vector. The Support Vector Machine (SVM) is then configured to build a multiple-subject classifier for which the subject independent accuracy will be given across all subjects for the classification of four types of lower-limb motions. In order to effectively indicate the classification performance, EMG features from time-domain (e.g., Mean Absolute Value (MAV), Root-Mean-Square (RMS), integrated EMG (iEMG), Zero Crossing (ZC)) and frequency-domain (e.g., Mean Frequency (MNF) and Median Frequency (MDF)) are also used to classify lower-limb motions. The five-fold cross validation is performed and it repeats fifty times in order to acquire the robust subject independent accuracy. Results show that the proposed WT-based SVD approach has the classification accuracy of 91.85%±0.88% which outperforms other feature models.

## 1 Introduction

The lower-limb motion is of vital importance in human daily living activities. It is frequently difficult for physically weak persons (i.e., elderly, disabled, and injured persons) to perform daily lower-limb activities. Recently, power-assisted robotic systems have been developed to target these problems and assist people who need help in terms of their daily living [1–6]. Most of these systems are originally activated by EMG signals as they can directly indicate the electrophysiological responses to daily living activities. Therefore, the study of EMG-based biofeedback and its relationship to lower-limb motions is significantly concerned with the control of power-assisted robotic systems [7–10].

A motor unit usually includes a motor neuron and the skeletal muscle fibres innervated by that motor neuron. The force of a muscle contraction is regulated by the number of activated motor units. The measured single-channel EMG signal is a superimposed electrical activity with the sum of activations from multiple motor units and a wide range of noise [11] [12]. For an exercise motion, the physiological behaviours from the brain activity are intrinsic [13]. In addition, the electric indications of the activated motor units for one muscle contraction should be unique, and only vary due to brain activity. The single-channel EMG signal relative to one particular muscle contraction is used to interpret the electrophysiological response from one perspective [14, 15]. In this study, a single-channel-based EMG pattern classification is developed in order to provide an easy-to-use condition for detections of lower-limb motions.

As described in detail previously [10], Agonist-antagonist muscles exist in human joints e.g., elbow, hip, wrist, knee, and ankle. These human joints are usually activated by either the biarticular or uniarticular muscles. For instance, a flexion/extension motion of human knee joint is actuated by the muscles of biceps femoris, semitendinossus, gastrocnemius, rectus femoris, vastus lateralis and vastus medialis. Most of these muscles are biarticular muscles except the uniarticular muscles of vastus lateralis and vastus medialis. In this study, the muscle of vastus medialis is used since it is primarily associated with muscle fatigue and has the significance for the human-machine interface [16].

The EMG signal is contaminated with various artifacts, and its time series data is not practical for classification purposes [17]. The conventional methods are to apply the time-dependent and the frequency-dependent properties to detect the EMG features across different knee exercise patterns [10, 18, 19], such as Mean Absolute Value (MAV), Root-Mean-Square (RMS), integrated EMG (iEMG), Zero Crossing (ZC)), Mean Frequency (MNF) and Median Frequency (MDF). Since EMG signals vary in frequency content over time, the conventional analysis methods cannot accurately describe its time-dependent statistical properties. In order to alleviate this problem, the following EMG feature extraction models of upper-limb activities (specific to wrist motions) have been developed including Fourier Transform (FT), Wavelet Transform (WT), Autoregressive (AR), Power Spectral Estimation (PSE), and Smooth Localised Complex Exponential (SLEX) [17, 20–23]. The recent review work in [17], where the classification accuracy rate of these feature vectors was evaluated by an Artificial Neural Network (ANN) classifier, clearly show that the time-frequency analysis methods have high accuracy rates, while the WT is much more efficient than the FT, as well as being better on the localisation of dominant subfrequency bands. In addition to Electroencephalogram (EEG) signals, it has been reported in previous study [24] that WT was employed to handle the EEGs and form a matrix, and then the singular value of the matrix is extracted. However, this method was limited to EMG-based classifications for lower limb activities.

The objective of this study is to propose the WT-based Singular Value Decomposition (SVD) approach for classification of EMG-based lower limb activities using a single-channel EMG signal from the muscle group of vastus medialis. Although WT-based approaches have been widely used for EMG signals, the combination of WT and SVD to comprise the feature vector for classifications of lower limb activities, are not well documented. In addition, the classification of those motions using only one single EMG channel is also nontrivial.

The experiment for data collections was approved by the University of Technology Sydney (UTS) Human Research Ethics Committee. An informed consent was obtained prior to data collection. The University of Electronic Science and Technology of China (UESTC) also approved to use the databases obtained from UTS for publications. The databases of 14 healthy untrained subjects are taken into the study. The subjects undergo three exercise programs associated with the knee joint, leg extension from a sitting position (sitting), flexion of the leg up (standing), and gait (walking), while EMG signals from the vastus medialis muscle of the detected leg are monitored and recorded. Based on the EMG datasets, four types of lower-limb motions including standing, sitting, stance phase of walking, and swing phase of walking, are segmented.

The Support Vector Machine (SVM) is then configured to build a multiple-subject classifier for which the subject independent accuracy will be given across all subjects for the classification of four types of lower-limb motions. In order to effectively indicate the classification performance, EMG features from time-domain and frequency-domain are also used to classify lower-limb motions. The five-fold cross validation is performed and it repeats fifty times in order to acquire the robust subject independent accuracy. By comparing outcomes from typical feature models with the one from the proposed WT-based SVD approach, the WT-based SVD approach provide best subject independent accuracy for standing, sitting, stance phase of walking, and swing phase of walking.

The remainder of the paper is organised as follows. Section ‘Method’ describes WT, SVD, EMG features, classification and evaluation procedure. Section ‘Experiment’ introduces experimental equipment, exercise procedures and protocols. Section ‘Results’ provides the outcomes from time-domain features, frequency-domain features, as well as the SVD-WT approach. Section ‘Discussions and Limitations’ clarifies the limitations and future directions of this study. Section ‘Conclusion’ concludes this study.

## 2 Methods

The University of Technology Sydney (UTS) Human Research Ethics Committee (UTS HREC 2009000227) approved this experiment and collected the EMG datasets. The University of Electronic Science and Technology of China (UESTC) also approved to use the databases obtained from UTS for publications. A written informed consent was obtained from every subject prior to the experiment. The University of Technology Sydney (UTS) Human Research Ethics Committee (UTS HREC 2009000227) approved this consent procedure.

### 2.1 Time-domain features

The Mean Absolute Value (MAV) is used to calculate the average of the absolute value of all time samples, which is essential for determining the baseline of single EMG channel during daily lower-limb motions. The equation is given by Eq (1)

$$\begin{array}{c} \bar{X}=\frac{1}{N}\sum_{k=1}^{N}\;\left|x_{k}\right|, \end{array}$$ (1)

where *x*_*k*_ is the potential at the k^*th*^ sampling and the parameter N is the number of samples [25].

The Root-Mean-Square (RMS) is also employed as a feature extraction method of the EMG signals in this study. The equation of RMS is written as Eq (2)

$$\begin{array}{c} RMS_{i}=\sqrt{\frac{1}{N}\sum_{k=1}^{N}\;x_{k}^{2}},\;\;for\;\;i=1,\ldots ,I \end{array}$$ (2)

where I is the number of segments.

The integrated EMG (iEMG) [26] is the mathematical integral of the absolute value of EMG normalized candidate segments, where can be expressed as Eq (3)

$$\begin{array}{c} iEMG_{i}=\sum_{k=1}^{N}\;\left|x_{k}\right|,\;\;for\;\;i=1,\ldots ,I. \end{array}$$ (3)

Zero Crossing (ZC) is a feature that tracks the number of times the waveform crosses zero, switching from a positive signal to a negative one, and vice versa [27], which was combined with the tone labels to detect the onset of movement during the procedure of data segmentation. Eq (4) is used for zero crossings

$$\begin{array}{c} \{\begin{array}{l} sgn\left(-x_{k}\times x_{k+1}\right)\\ |x_{k}-x_{k+1}|\geq threshold \end{array} \end{array}$$ (4)

where the sgn function returns a positive number if the threshold is exceeded, and the zero crossing count is incremented [27, 28].

### 2.2 Frequency-domain features

Mean Frequency (MNF) [29] is the average frequency of an EMG segment representing the lower-limb motion. MNF is calculated as the sum of the product of the amplitude spectrum and the frequency, divided by the total sum of spectrum intensity. The mathematic expression can be written as Eq (5)

$$\begin{array}{c} MNF_{i}=\sum_{j=1}^{M}\;P_{ij}f_{ij}/\sum_{j=1}^{M}\;P_{ij},\;\;for\;\;i=1,\ldots ,I \end{array}$$ (5)

where *P*_*ij*_ is the EMG power spectrum at the frequency bin *j* for the *i*th segment, *f*_*ij*_ is the frequency value of EMG power spectrum at the frequency bin *j* for the *i*th segment, and *M* is the length of frequency window.

Median Frequency (MDF) is the frequency at which the spectrum is divided into two regions with equal amplitude. It can be calculated in the following two steps: [29]

a). The signal intensities in the whole spectrum are summed and then divided by two (see Eq (6)),

$$\begin{array}{c} MDF_{i}=\frac{1}{2}\sum_{j=1}^{M}\;P_{ij},\;\;for\;\;i=1,\ldots ,I. \end{array}$$ (6)

b). The MDF frequency is selected at which the cumulative intensity first exceeds *MDF*_*i*_.

### 2.3 Five-level wavelet decomposition

The frequency of human EMG signal is usually between 10–500 Hz [30, 31]. WT is a time-frequency tool to analyse EMG signals. It can decompose signals into different scales and provide more information from time and frequency domains. In this paper, a five-level wavelet decomposition is employed by using Daubechies4 algorithm [32]. Based on the decomposition of EMG signals, the restricted frequency bandwidth for the components from cD1, cD2, cD3, cD4, cA5 are 256 ∼ 512 Hz, 128 ∼ 256 Hz, 64 ∼ 128 Hz, 32 ∼ 64 Hz, and 16 ∼ 32 Hz, respectively. cA*n*(*n* = 1, 2, …, 5) is the coefficients for low-frequency components of the signals. cD*n* is the coefficients for the high-frequency components. At each level, only one parameter from cD*n* can be retained according to the SVD method which can compress cD*n* to one parameter.

#### 2.3.1 Singular Value Decomposition (SVD)

According to Section 2.3, the EMG data of four lower-limb motions above are decomposed by WT, and feature matrices (*M*_*ij*_ = [*cD*_1*i*_; *cD*_2*i*_; *cD*_3*i*_; *cD*_4*i*_; *cD*_5*i*_; *cA*_5*i*_], *i* = 1, 2, …, *I*) will be built. In order to pursue the simple and effective feature vector for the following classification by SVM, the singular value of *M*_*ij*_ will be obtained by SVD.

Let A, B ∈ *C*^*m*×*n*^, if there are *m* order unitary matrix U and *n* order unitary matrix V, *U*^*T*^ *AV* = *B*, then A and B are unitary equivalent. If A $\in C_{r}^{m\times n}$(r > 0), the eigenvalues of *A*^*T*^ *A* should have the following relation equation as Eq (7)

$$\begin{array}{c} \lambda_{1}\geq \lambda_{2}\geq \cdots \geq \lambda_{r}\geq \lambda_{r+1}=\cdots =\lambda_{n}=0, \end{array}$$ (7)

then $\sigma_{i}=\sqrt{\lambda_{i}}\left(i=1,2,\ldots ,n\right)$ are the singular values of A.

If $\text{A}\in C_{r}^{m\times n}(\text{r}>0)$, then there must be *m* order unitary matrix U and n order unitary matrix V, they satisfy Eq (8)

$$\begin{array}{c} U^{T}AV=[\begin{array}{cc} \sum \;\; & \;\;0\\ 0\;\; & \;\;0 \end{array}] \end{array}$$ (8)

where ∑ = *diag*(*σ*_1_, *σ*_2_, …, *σ*_*r*_), and *σ*_*i*_(*i* = 1, 2, …, *r*) are the nonzero singular values of A. Eq (8) can be transformed to Eqs (9), (10) and (11)

$$\begin{array}{c} A=U\;[\begin{array}{cc} \sum \;\; & \;\;0\\ 0\;\; & \;\;0 \end{array}]V^{T} \end{array}$$ (9)

where

$$\begin{array}{c} U=AA^{T} \end{array}$$ (10)

$$\begin{array}{c} V=A^{T}A \end{array}$$ (11)

Eq (9) is called the SVD of A.

### 2.4 Support vector regression

Let ${\left\{u_{i},y_{i}\right\}}_{i=1}^{N}$ be a set of inputs and outputs data points (*u*_*i*_ ∈ *U* ⊆ *R*^*d*^, *y*_*i*_ ∈ *Y* ⊆ *R*, N is the number of points). The goal of the support vector regression is to find a function *f*(*u*) which has the following form

$$\begin{array}{c} f(u)=w\cdot \phi (u)+b, \end{array}$$ (12)

where *ϕ*(*u*) is the high-dimensional feature spaces which are nonlinearly transformed from *u*. The weight vector *w* and bias *b* are defined as the hyperplane by the equation 〈*w* ⋅ *ϕ*(*u*)〉 + *b* = 0. The hyperplane is estimated by minimizing the regularized risk function

$$\begin{array}{c} \frac{1}{2}{∥w∥}^{2}+C\;\frac{1}{N}\;\sum_{i=1}^{N}\;L_{\varepsilon }\left(y_{i},f\left(u_{i}\right)\right), \end{array}$$ (13)

The first term is called the regularized term. The second term is the empirical error measured by *ε*-insensitivity loss function which is defined as

$$\begin{array}{c} L_{\varepsilon }\left(y_{i},f\left(u_{i}\right)\right)=\{\begin{array}{cc} |y_{i}-f\left(u_{i}\right)|-\varepsilon , & |y_{i}-f\left(u_{i}\right)|>\varepsilon \\ 0, & |y_{i}-f\left(u_{i}\right)|\leq \varepsilon \end{array} \end{array}$$ (14)

This defines an *ε* tube. The radius *ε* of the tube and the regularization constant *C* are both determined by user.

The selection of parameter *C* depends on application knowledge of the domain. Theoretically, a small value of *C* will under-fit the training data because the weight placed on the training data is too small, thus resulting in large values of MSE (mean square error) on the test sets. However, when *C* is too large, SVR will over-fit the training set so that $\frac{1}{2}{∥w∥}^{2}$ will lose its meaning and the objective goes back to minimize the empirical risk only. Parameter *ε* controls the width of the *ε*-insensitive zone. Generally, the larger the *ε* the fewer number of support vectors and thus the sparser the representation of the solution. However, if the *ε* is too large, it can deteriorate the accuracy on the training data.

By solving the above constrained optimization problem, we have

$$\begin{array}{c} f\left(u\right)=\sum_{i=1}^{N}\;\beta_{i}\phi \left(u_{i}\right)\cdot \phi \left(u_{i}\right)+b, \end{array}$$ (15)

As mentioned above, by the use of kernels, all necessary computations can be performed directly in the input space, without having to compute the map *ϕ*(*u*) explicitly. After introducing kernel function *k*(*u*_*i*_, *u*_*j*_), the above equation can be rewritten as follows

$$\begin{array}{c} f\left(u\right)=\sum_{i=1}^{N}\;\beta_{i}k\left(u_{i},u\right)+b, \end{array}$$ (16)

where the coefficients *β*_*i*_ corresponding to each (*u*_*i*_, *y*_*i*_). The support vectors are the input vectors *u*_*j*_ whose corresponding coefficients *β*_*j*_ ≠ 0. For linear support regression, the kernel function is thus the inner product in the input space

$$\begin{array}{c} f\left(u\right)=\sum_{i=1}^{N}\;\beta_{i}\left\langle u_{i},u\right\rangle +b, \end{array}$$ (17)

For nonlinear SVR, there are a number of kernel functions which have been found to provide good generalization capabilities, such as polynomials, radial basis function (RBF), sigmod. Here we present the polynomials and RBF kernel functions as follows:

Polynomial kernel: *k*(*u*, *u*′) = ((*u* ⋅ *u*′) + *h*)^*p*^.

RBF Kernel: $k\left(u,u^{\prime }\right)=exp\left(-\frac{∥u-u^{\prime }{∥}^{2}}{2\sigma^{2}}\right)$.

Details about SVR, such as the selection of radius *ε* of the tube, kernel function, and the regularization constant *C*, can be found in [33–36]. The origin of the SVM code applied in this study was obtained from [37].

### 2.5 Classification

For the classification of four knee exercises, the singular values of these matrices decomposed by WF, *M*_*ij*_, are calculated as the time-frequency features of EMG signals in the vastus medialis muscle. For the selected candidate segments, the formula is

$$\begin{array}{c} M_{ij}=[\begin{array}{cccc} \sigma_{11} & \sigma_{12} & \ldots & \sigma_{1j}\\ \sigma_{21} & \sigma_{22} & \ldots & \sigma_{2j}\\ \vdots & \vdots & \vdots & \vdots \\ \sigma_{i1} & \sigma_{i2} & \ldots & \sigma_{ij} \end{array}] \end{array}$$ (18)

where i is the number of singular values for each trial data, and j is the number of EMG candidate segments.

The values of RMS and iEMG (see Eqs (2) and (3), respectively) of the time-domain based EMG signals are chosen as the time-domain features. In addition, the frequency based characteristics inclusive of the values of MNF and MDF (see Eqs (5) and (6), respectively) across all trials are calculated as the frequency-domain features.

For the testing sample, x, the discriminant function is

$$\begin{array}{c} f\left(x\right)=\sum_{i=1}^{N_{s}}\;y_{i}\alpha_{i}G\left(x,x_{i}\right)+b \end{array}$$ (19)

where *N*_*s*_ is the number of the resulting support vectors, *α*_*i*_ is the positive Lagrangian multipliers and *G*(*x*, *x*_*i*_) is a kernel function. The vital regularization parameter in SVM is also determined by the five-fold cross-validation procedure that is repeated fifty times in order to avoid the random errors.

### 2.6 Evaluation procedure

**Fig 1:**
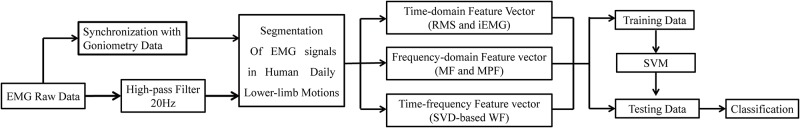
The evaluation procedure for classification of four types of lower-limb motions.

In order to indicate the classification performance in terms of standing, sitting, stance phase of walking, and swing phase of walking, features from the time domain, the frequency domain, and the WT-based SVD approach are tested separately through the predefined evaluation procedure shown in Fig 1. In the procedure, the raw EMG data is first filtered by a high-pass filter with 20 Hz. The signals then are trial-by-trail segmented following the synchronized goniometry data. After preprocessing and segmentation of the raw EMG signals, the features represented by the analyses of the time domain, the frequency domain, and the WT-based SVD approach are extracted to build the feature vectors. A SVM-based multiple subject classifier was trained by the trials across all subjects and the trained classifier was then used for predicting and accumulating of the discrimination evidence over features. The datasets of four types of lower-limb motions from all subjects were randomly shuffled and then partitioned totally five times. It then was divided into a training set (80% of dataset) or a testing set (20% of dataset). The five-fold cross validation is used for the method evaluations and it is repetitively run fifty times to avoid the random errors. The average of the total accuracy results for fifty-time five-fold cross-validations is computed as the final accuracy result.

## 3 Experiment

The University of Technology Sydney (UTS) Human Research Ethics Committee (UTS HREC 2009000227) approved this experiment and collected the EMG datasets. A poster was used to introduce this experiment in order to recruit the participants from UTS campus, which was publicly open during the summer holiday in 2012. An informed consent was obtained for each participant prior to data collection. After the experiment, the remuneration was paid to the participants. The University of Electronic Science and Technology of China (UESTC) also approved to use the databases obtained from UTS for publications.

The EMG data collection process was carried out with one electrode placed on the muscle group of vastus medialis and the goniometer on the knee joint. The Datalog device MWX8 (http://www.biometricsltd.com/datalog.htm) is used for EMG data acquisition including 8 digital channels and 4 analog channels. One of the digital channels is used for monitoring and recording EMG signals and another for goniometry data of the knee joint of the detected foot. The data are first acquired and memorized in the MWX8 internal storage with microSD card, and then transmitted through the real-time Datalog software via Bluetooth adapter for the offline analyses for this study. The sampling frequency is 1 kHz [38].

Since the EMG signals repetitively obtained from one subject can vary from the emotional state, food and caffeine intake, previous activity, fatigue, only healthy and untrained subjects were invited in this experiment. Moreover, the participants were asked to have a light meal at least two hours before the experiment and not to engage in intense or prolonged exercise for 24 hours prior to each experiment. Environmental conditions were the same for all participants.

In order to minimize the impedance of the electrode-skin contact in-between, the hair and dead skin cells on the target electrode placement location were shaved from the skin surface before prior to experiment. Once shaved, the skin was cleaned with alcohol and the electrode is then placed on the target skin after it comes to dry. The bipolar electrode (SX230) is used for the experiment. A fixed electrode distance of 20 *mm* is offered. It offers an integral electrode with fixed inter-electrode distance which gives consistent high quality results and effectively limits the risk of inter-electrode cross-talk. The R506 compatible with Datalog device MWX8 has a standard 4*mm* snap connector, which could minimize the risk of simultaneously recording similar signals. The input impedance of the EMG amplifier is more than 10,000,000 M Ohms [38].

**Fig 2:**
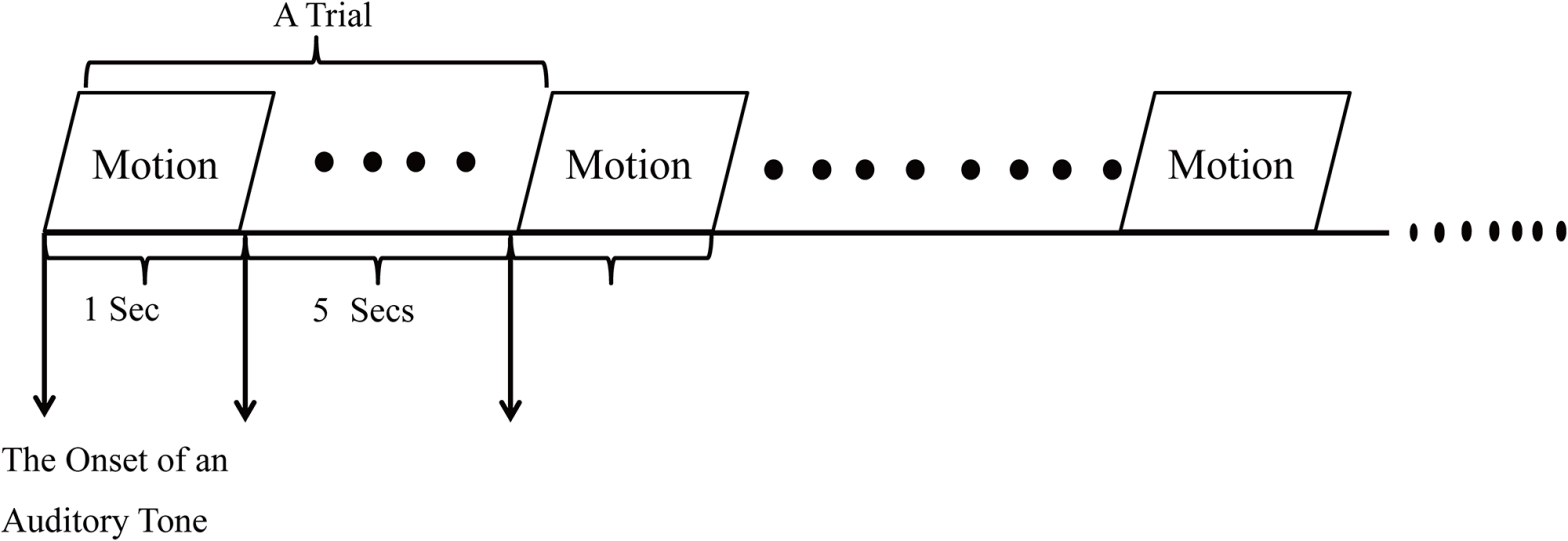
The experiment protocol for EMG data collection in terms of standing-sitting and walking sessions.

The databases of 14 healthy untrained subjects (age: 28±7.82 years old, height: 174±5.88 cm, mass: 67.64±11.35 kg) are taken into the study. The subjects undergo three exercise programs associated with the knee joint, leg extension from a sitting position (sitting), flexion of the leg up (standing), and gait (walking), while EMG signals from the vastus medialis muscle of the detected leg are monitored and recorded. Two experimental sessions are arranged, i.e., standing-sitting and walking, in which subjects are asked to randomly perform these exercise programs. For both sessions, subjects are asked to start the onset of motions with a one-second tone-paced timing with one-second interval, and follow a rest with five-second interval. Each motion is repeated five times in order to avoid intra-subject variability. For the standing-sitting motion, the order of performing both motions is randomly chosen. In addition, a fifteen-minute break is offered between two sessions. The details of exercise protocol are as follows in Fig 2. During the experiments, a tone-paced timing is presented which follows an automatic tone, pacing interval where the onset of each motion is matched with the onset of an auditory tone in order to remind subject to start and stop their motions. Based on the EMG datasets obtained from both sessions, four types of lower-limb motions including standing, sitting, stance phase of walking, and swing phase of walking, are segmented, which will be used for the classification evaluation of the proposed WT-based SVD approach. For the details of original data, see S1 File.

## 4 Results

### 4.1 Time-domain analyses

**Fig 3:**
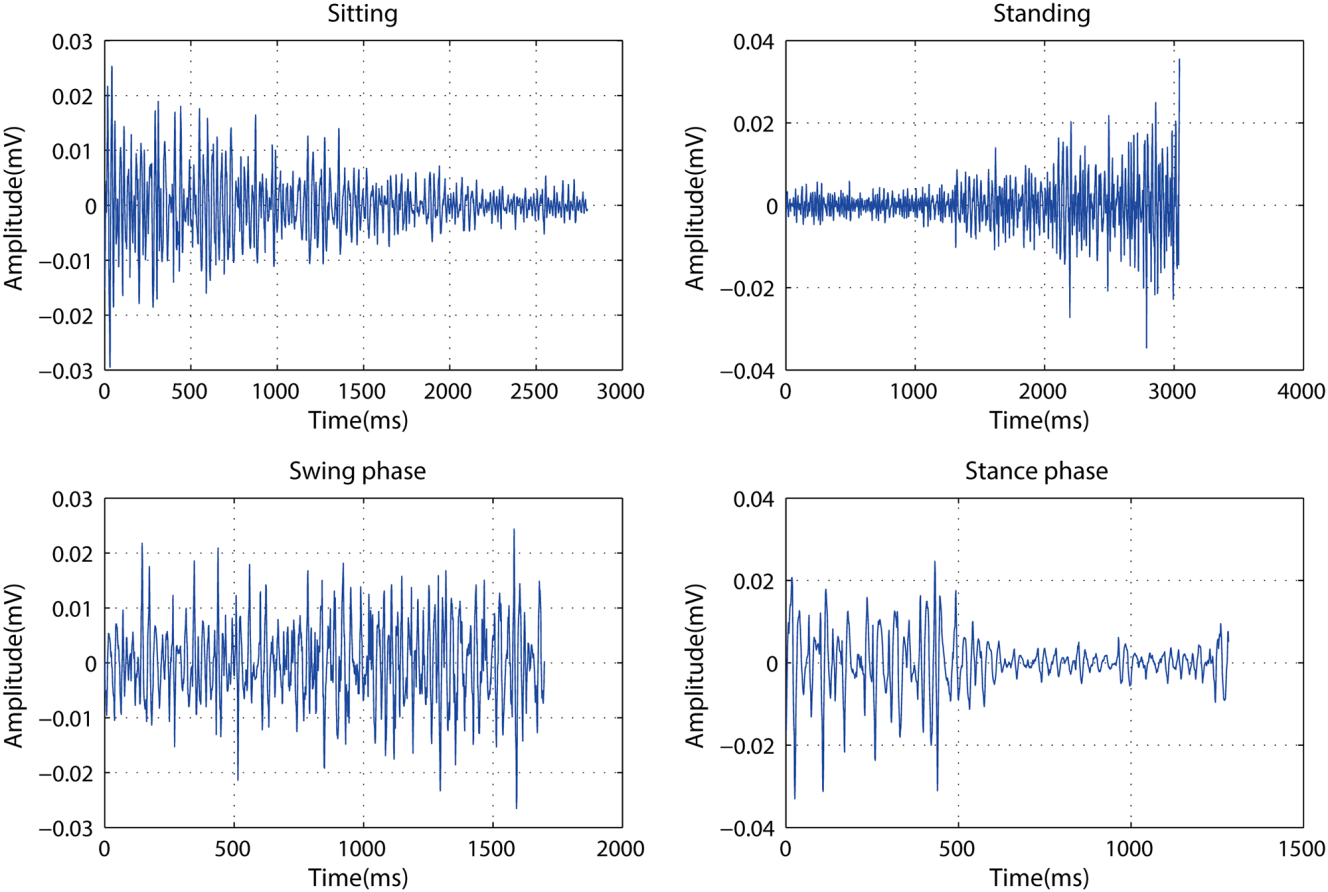
The EMG time series for the segmented lower-limb motions in terms of the swing phase, the stance phase, sitting, and standing.

In the preprocessing step, the raw EMG data is filtered by using a 20 Hz butterworth high-pass filter. The filtered data is then segmented following the synchronized goniometry data. The segmented trials include lower-limb motions for standing, sitting, stance phase of walking, and swing phase of walking. The EMG time series of such types of trails are presented in Fig 3. It can be found that for both standing and sitting motions the EMG strength increases (decreases) along with the process of flexion (extension) of the detected leg; for swing and stance phases the variations of the amplitudes of the swing phase are less affected compared with those of the stance phase.

The time-domain features for each trial across all subjects including MAV, RMS, iEMG, and ZC, are calculated. Table 1 shows the mean and STD values of those time-domain features for four lower-limb motions. Based on the segmented EMG data for all trials, the statistical analysis for these four time-domain features is evaluated by one-way analysis of variance analysis, which shows that MAV, RMS, iEMGP, and ZC have means significantly different for lower-limb motions (standing, sitting, stance phase of walking, and swing phase of walking) (p < 0.05).

**Table 1:** The mean and STD for temporal features based on experimental trails from all participants on the vastus medialis muscle.

	MAV Mean±STD%	RMS Mean±STD%	iEMG Mean±STD%	ZC Mean±STD%
Swing	0.02±50	0.03±33.3	25.42±45.25	137.03±35.88
Stance	0.01±100	0.02±50	27.28±78.85	260.82±22.11
Standing	0.01±200	0.01±400	5.44±143.38	97.84±73.95
Sitting	0.01±300	0.02±300	11.65±188.24	132.82±56.99

STD% represents the percentage of the standard deviation over the corresponding mean.

### 4.2 Frequency-domain analyses

**Fig 4:**
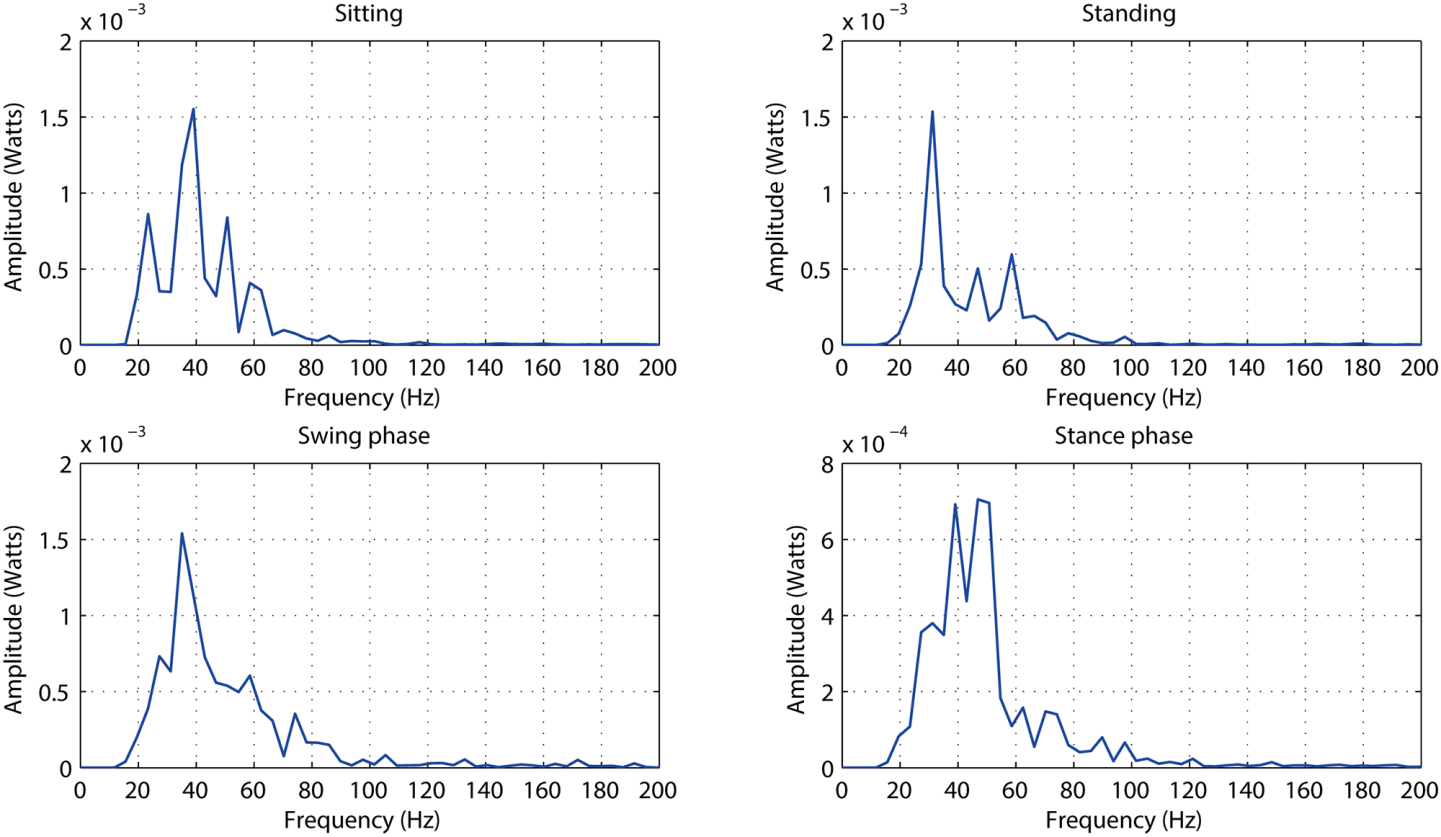
The PSD curves of the segmented EMG time series in terms of the swing phase, the stance phase, sitting, and standing.

In this study, the frequency-domain features of EMG segmentations are also investigated. The raw data is filtered by 20 Hz high-pass filter and then segmented following the synchronized goniometry data in order to obtain the specified EMG signals for lower-limb motions. The Welch’s averaged periodogram method [39] is utilized to demonstrate the Power Spectrum Density (PSD) of the EMG segmented data. The PSD curves based on the different types of lower-limb motions from one subject are shown in Fig 4.

The frequency-domain features such as MNF and MDF are calculated based on each trail data. The averaged MNF and MDF results for all trials of each motions across all subjects are provided in Table 2. Based on the calculated MNF and MDF values for all trials, the statistical analysis for the frequency-domain features is evaluated by one-way analysis of variance analysis, which shows that MNF and MDF have means significantly different for lower-limb motions (standing, sitting, stance phase of walking, and swing phase of walking) (p < 0.05). Furthermore, paired t-test results indicate that there are significant differences between any two of the four lower-limb motions for the calculated MNF and MDF values. Therefore, both MDF and MNF are used to represent the frequency-domain feature.

**Table 2:** The mean and STD for frequency-domain features based on experimental trails from all participants on the vastus medialis muscle.

	MDF Mean±STD%	MNF Mean±STD%
Swing phase	45.43±11.64	53.07±9.37
Stance phase	48.48±13.51	54.17±7.72
Standing	36.52±22.78	43.82±19.17
Sitting	38.38±22.64	45.25±19.51

STD% represents the percentage of the standard deviation over thecorresponding mean. The unit for mean values of MDF and MNF is Hz.

### 4.3 Time-frequency features extracted by WT-based SVD analyses

In order to obtain the time-frequency features by using the proposed WT-based SVD approach, the raw EMG data are also preprocessed through a 20 Hz high-pass filter. The filtered EMG data are then segmented to establish the candidate datasets for classifications. As mentioned in Section ‘Methods’, the five-level wavelet decomposition is applied to decompose the candidate EMG segmentations into five different scales time series. The coefficients for the high-frequency components (cD1, cD2, cD3, cD4, and cD5) and the coefficient for the low-frequency component at the fifth level (cA5) are given. The singular value of each components is then computed by SVD. Table 3 shows the mean and STD values of the singular values for each decomposed components. Based on these results, an one-way analysis of variance analysis is also used, by which the statistical analysis results indicate that the six components have means significant difference for lower-limb motions (standing, sitting, stance phase of walking, and swing phase of walking) (p < 0.05).

**Table 3:** The mean and STD results of time-frequency features by WT-based SVD approach based on experimental trails from all participants on the vastus medialis muscle.

	Stance Mean±STD%	Swing Mean±STD%	Standing Mean±STD%	Sitting Mean±STD%
cD1	0.22±68.18	0.25±48	0.26±430.77	0.26±315.38
cD2	0.44±40.91	0.35±71.43	0.23±213.64	0.48±274.47
cD3	0.85±40	0.65±78.46	0.24±216.67	0.4±231.71
cD4	0.54±62.96	0.42±66.67	0.1±150	0.16±168.75
cD5	0.21±55	0.16±68.75	0.04±125	0.06±150
cA5	0.11±70	0.08±75	0.02±100	0.03±100

STD% represents the percentage of the standard deviation over the corresponding mean.

### 4.4 Classification results

Based on the proposed evaluation procedure, the SVM is used to build a multiple-subject classifier for which the subject independent accuracy will be given across all subjects for the classification of four types of lower-limb motions. The five-fold cross validation is performed and it repeats fifty times in order to acquire the robust subject independent accuracy.

There were 260 EMG trials collected from three subjects participating in the lower-limb motion experiment, and for all subjects they were set into four sets of 40 segments for standing, 40 for sitting, 90 for the swing phase and 90 for the stance phase. The datasets are then randomly partitioned into five equal sized subsets. Of the five subsets, a single subset is retained as the validation data for testing, and the remaining four subsets are used for training. The evaluation procedure is then repeated fifty times, with each of the five subsets used exactly once as the validation data. The averaged results from the fold are then used to produce the subject independent accuracy across all subjects’ trails.

Three different features are used as the feature vectors to train the SVM-based multiple-subject classifier, i.e., time-domain features (MAV, RMS, iEMG, and ZC), frequency-domain feature (MNF+MDF), and WT-based SVD features (cD1, cD2, cD3, cD4, cD5, and cA5). In order to evaluate the classification performance, there are five different feature vectors established for training and testing the lower-limb motions, including time-domain features (MAV+RMS+iEMG+ZC), frequency-domain feature (MNF+MDF), combined time and frequency features (MAV+RMS+iEMG+ZC+MDF+MNF), WT-based SVD features (cD1+cD2+cD3+cD4+cD5+cA5), and combined time, frequency, and WT-based SVD features (MAV+RMS+iEMG+ZC+MDF+MNF+cD1+cD2+cD3+cD4+cD5+cA5). The classification results are demonstrated in Table 4

**Table 4:** The subject independent accuracies for standing, sitting, stance phase of walking, and swing phase of walking.

Feature vectors	Accuracy(%)	STD(%)
MAV+RMS+iEMG+ZC	66.96	3.12
MNF+MDF	77.1	0.01
MAV+RMS+iEMG+ZC+MDF+MNF	55.11	0.02
cD1+cD2+cD3+cD4+cD5+cA5	**91.85**	**0.88**
MAV+RMS+iEMG+ZC+MDF+MNF+cD1+cD2+cD3+cD4+cD5+cA5	53.36	0.02

STD represents the percentage of the standard deviation over the corresponding accuracy.

**Fig 5:**
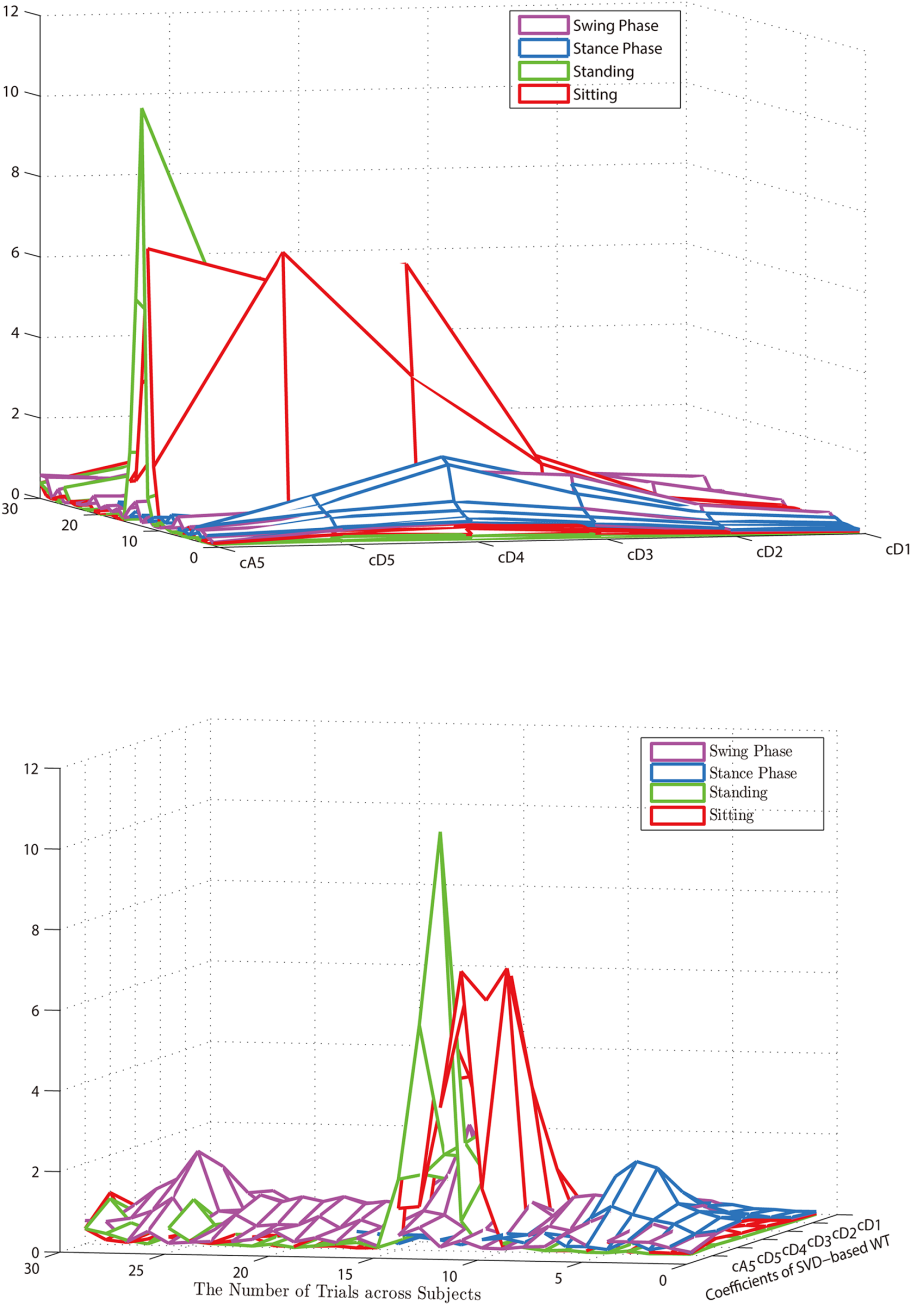
The WT-based SVD features for all trials with four lower-limb motions.

The WT-based SVD features are demonstrated in Fig 5, where the WF-based SVD coefficients for all trials with four lower-limb motions are plotted. Based on this feature vector, the classification results show that the proposed WT-based SVD approach has the classification accuracy of 91.85%±0.88% which outperforms the time-domain features (66.96%%±3.12%), the frequency-domain features (77.1%%±0.01%), the combined time and frequency features (55.11%%±0.02%), and the combined time, frequency, and WT-based SVD features (53.36%%±0.02%).

## Discussions and limitations

It has been clearly shown that either time-domain features or frequency-domain features cannot completely depict the differences among those motions. Results also confirmed that the combined features (combined time and frequency features and combined time, frequency, and WT-based SVD features) also cannot give a better classification accuracy compared to the WT-based SVD features. It is the fact that the combined features cannot maximize the discriminative power of EMG features for classifying lower-limb motions with a single EMG channel placed on the vastus medialis.

The contribution of this work is to introduce the SVD-based WT approach for the single-channel-based classification of lower limb activities. In the first step, it was natural to first explore the recognition accuracy of such proposal. In order to find discriminative features, the statistical analysis was employed in this study. The databases of 14 healthy untrained subjects are taken into the study. The subjects undergo three exercise programs associated with the knee joint, leg extension from a sitting position (sitting), flexion of the leg up (standing), and gait (walking), while EMG signals from the vastus medialis muscle of the detected leg are monitored and recorded. Based on the EMG datasets obtained from both sessions, four types of lower-limb motions including standing, sitting, stance phase of walking, and swing phase of walking, are segmented, which will be used for the classification evaluation of the proposed WT-based SVD approach.

Fig 3 explicitly shows that the amplitudes between sitting, standing stance phase of walking, and swing phase of walking, are distinctively different. The multivariate analysis is utilized by the one-way analysis of variance where the time-domain features, MAV, RMS, iEMG and ZC, have means significantly different. Thus, those are used for the analysis of time features in relation to knee exercise pattern recognition. Secondly, Mean Frequency (MNF) and Median Frequency (MDF) are used to indicate the frequency-domain characteristics of the EMG trails (see Fig 4). Based on these statistical results, MNF is selected as frequency-domain features due to the significant difference between any two of lower-limb motions.

Based on the classification results, it has been clearly shown that either time-domain features or frequency-domain features cannot completely depict the differences among those motions. Following this reason, the time-frequency features extracted by using the proposed SVD-WT approach are used in this study. The results present time-frequency features by using SVD-WT outcomes is better than ones by using either time-domain features or frequency-domain features (see Fig 5 and Table 4). However, this study has not addressed an optimal feature model described by the specified wavelet components that might achieve a better accuracy performance compared to the feature vectors with all wavelet components. In the next step of this study, Fisher-Markov selector will be applied in this study and quantitatively optimize the proposed WT-based SVD feature model [40] [41].

## Conclusion

In this study, the single-channel EMG signals from the muscle group of vastus medialis were recorded. 14 subjects participated in the three exercise programs, while EMG signals from the muscle group of vastus medialis and the goniometer on the knee joint of the detected leg were synchronously monitored and recorded. Four types of lower-limb motions including standing, sitting, stance phase of walking, and swing phase of walking, were segmented. Based on the experimental data, the SVM is then configured to build a multiple-subject classifier for which the subject independent accuracy will be given across all subjects for the classification of four types of lower-limb motions. In order to effectively indicate the classification performance, EMG features from time-domain (e.g., Mean Absolute Value (MAV), Root-Mean-Square (RMS), integrated EMG (iEMG), Zero Crossing (ZC)) and frequency-domain (e.g., Mean Frequency (MNF) and Median Frequency (MDF)) were also used to classify lower-limb motions. The five-fold cross validation was performed and it repeats fifty times in order to acquire the robust subject independent accuracy. Three different features were used as the feature vectors to train the SVM-based multiple-subject classifier, i.e., time-domain features, frequency-domain feature, and WT-based SVD features. In order to evaluate the classification performance, there are five different feature vectors established for training and testing the lower-limb motions, including time-domain features (MAV+RMS+iEMG+ZC), frequency-domain feature (MNF+MDF), combined time and frequency features (MAV+RMS+iEMG+ZC+MDF+MNF), WT-based SVD features (cD1+cD2+cD3+cD4+cD5+cA5), and combined time, frequency, and WT-based SVD features (MAV+RMS+iEMG+ZC+MDF+MNF+cD1+cD2+cD3+cD4+cD5+cA5). Based on this feature vector, the classification results show that the proposed WT-based SVD approach has the classification accuracy of 91.85%±0.88% which outperforms other feature models.

## Supporting information

S1 File Original EMG datasets. The EMG orignial data of the vastus medialis muscle of the detected leg from 14 healthy subjects were collected and recorded in the file while performing three exercise programs, i.e., leg extension from a sitting position (sitting), flexion of the leg up (standing), and gait (walking).
(ZIP)
